# 肺癌合并严重冠心病同期外科手术治疗经验

**DOI:** 10.3779/j.issn.1009-3419.2012.10.07

**Published:** 2012-10-20

**Authors:** 旭晨 马, 志泰 张, 燕生 胡, 飞强 宋, 韶岩 张, 颂雷 区

**Affiliations:** 100029 北京，首都医科大学附属北京安贞医院胸外科 Department of Thoracic Surgery, Beijing An-zhen Hospital Attached to the Capital University of Medical Science, Beijing 100029, China

**Keywords:** 肺肿瘤, 冠心病, 手术, Lung neoplasms, Coronary heart disease, Surgery

## Abstract

**背景与目的:**

临床上肺癌合并严重冠心病患者逐年增多，本文旨在总结同期心肺手术治疗这类患者的临床经验，为临床诊治工作提供参考。

**方法:**

回顾性分析2003年-2012年我科完成18例同期手术的临床资料。男性16例，女性2例，平均年龄66.11岁。临床TNM分期多为Ⅰ期/Ⅱ期病例，心肺功能良好。

**结果:**

本组病例无手术死亡及新发心肌梗塞。病理诊断鳞癌10例，腺癌8例，病理TNM分期Ⅰa期2例，Ⅰb期8例，Ⅱa期3例，Ⅱb期3例，Ⅲa期2例。术后常见并发症为心律失常、肺不张、肺部感染。手术时间、术后引流量、带管时间及输血量腔镜组均较开胸组有明显下降，两组生存率无统计学差异（*P*=0.187）。

**结论:**

同期手术治疗肺癌合并严重冠心病安全、有效。胸腔镜使用创伤小，效果较确切。

## Abstract (en)

**Background and objective:**

The number of patients with both lung cancer and coronary heart disease has increased for the last ten years. The aim of this study is to analyze the outcome of the combined treatment of radical surgery and off-pump coronary artery bypass grafting (OPCABG) for patients with both lung cancer and coronary heart disease.

**Methods:**

The clinical data of 18 patients (16 males and 2 females, mean age of 66.11 years) with both lung cancer and coronary heart disease who went through combined surgical interventions between 2003 and 2012 were summarized and analyzed. The lung cancer in these patients was predominantly at stages Ⅰ and Ⅱ (TNM). All patients' cardio-pulmonary function was favorable.

**Results:**

All the patients survived the operation, without any recorded death and new myocardial infarction occurrence during the perioperative period. Squamous carcinoma and adenocarcinoma were found in 10 and 8 patients, respectively. Pathological lung cancer stage was Ⅰa in 2 cases, Ⅰb in 8 cases, Ⅱa in 3 cases, Ⅱb in 3 cases, and Ⅲa in 2 cases. The most frequently observed complications were cardiac arrhythmias, atelectasis, and pulmonary infections. The mean values of operating room time, postoperative drainage, drainage tube time, and blood transfusion were significantly different between video-assisted thoracoscopic surgery (VATS) groups and thoractomy groups. No significant differences in survival rate were found (*P*=0.187).

**Conclusion:**

The combined method of OPCABG and pulmonary resection is a safe and effective treatment for patients with both lung cancer and coronary heart disease. VATS lobectomy is beneficial for lung cancer patients because it reduces lesions.

近二十年肺癌及冠心病的发病率在我国逐年增加，城市中肺癌及冠心病已成为威胁人们健康的重要疾病。每年约有上百万患者死于这两类疾病。国外文献^[1, 2]^报道约10%的肺部肿瘤患者合并不同程度的心肌缺血症状。为了改善患者心肌缺血症状又不延误肺癌的外科治疗，首都医科大学附属北京安贞医院胸外科2003年-2011年共完成不停跳非体外循环下冠脉搭桥同期肺癌根治手术18例。现总结报道如下。

## 1 材料与方法

### 1.1 临床材料

2003年-2011年共完成18例同期肺癌根治及不停跳冠状动脉旁路移植术，男性16例，女性2例，年龄54岁-77岁，平均年龄66.11岁±5.75岁。冠状动脉病变以2支或3支血管狭窄为主（狭窄程度均 > 70%），心功能分级为Ⅰ级/Ⅱ级（NYHA分级标准）。临床肺部肿瘤TNM分期多为Ⅰ期/Ⅱ期病例。肺功能、动脉血气分析、超声心动图检查心肺功能未见明显异常。脑核磁、全身骨扫描、腹部超声检查未见远处转移。患者同期手术适应症采用相同标准，整体病例相似。18例患者经正中切口完成不停跳搭桥手术，平均搭桥2.3根，约1/3的患者使用乳内动脉。搭桥完成后胸腔镜辅助原切口行肺癌根治术7例, 侧翻身后外侧胸切口完成肺癌手术11例。

### 1.2 同期手术步骤

静脉复合麻醉，双腔气管插管后正中劈开胸骨，经患侧胸膜腔先行肺部肿瘤局部切除或活检，行快速冰冻明确病理诊断。肝素1 mg/kg剂量抗凝，游离健侧乳内动脉及下肢大隐静脉行冠脉搭桥手术，鱼精蛋白对抗肝素。18例患者平均搭桥2.3根，约1/3的患者使用乳内动脉。冠脉旁路移植完成后，根据肺部活检的病理结果实施肺癌根治手术。本组早期病例采用改变患者体位，重新消毒，后经改良为外侧胸部小切口行肺叶切除及淋巴结清扫11例，后期病例在胸腔镜辅助下经正中单一切口完成肺癌根治7例。具体术式见表 1。

**1:** 具体肺切除术式 The details of the type and the number of lung resections

Lung resection type	Video-assisted thoracoscopic surgery (VATS) group (*n*=7)	Thoractomy group (*n*=11)
Lobectomy (*n*=14)	5	9
Right upper	1	2
Right lower	2	2
Right middle	1	0
Left upper	0	2
Left lower	1	3
Bilobectomy (*n*=2)	0	2
Left upper	0	1
Left lower	0	1
Segment resection (*n*=2)	2	0
Left upper	2	0

### 1.3 统计方法

利用SPSS 13.0软件进行数据分析，采用独立样本*t*检验对胸腔镜手术和非胸腔镜手术两组的住院时间、ICU时间、手术时间、输血量、胸腔引流量及带管时间等方面进行比较。所有患者定期随诊，采用*Kaplan-Meier*方法分析两组生存情况。*P* < 0.05为差异有统计学意义。

## 2 结果

所有患者均安全完成同期冠脉搭桥及肺切除手术，围手术期无死亡及新发心肌梗塞情况。病理诊断鳞癌10例，腺癌8例；TNM分期Ⅰa期2例，Ⅰb期8例，Ⅱa期3例，Ⅱb期3例，Ⅲa期2例。本组病例总平均住院天数为（17.16±6.76）天，平均ICU时间是（14.83±4.13）h。手术时间平均为（208.33±44.62）min。术后胸腔及纵隔引流管平均呆管时间为（4.50±.92）天，引流量为（708.33±229.81）mL。术后常见合并症是心律失常、肺不张、肺部感染。胸腔镜组发生率为42.85%（房颤2例，肺部感染1例），常规开胸组为54.55%（房颤2例，室性心律失常1例，肺不张2例，感染1例）。对症处理均痊愈。腔镜组在手术时间、术后引流量、输血量及带管时间均较开胸组下降，而住院及ICU时间等方面两组无统计学差异，可能和肺切除与搭桥手术同期实施，数据无法分割有关。术后两组具体临床结果数据见表 2。

**2:** 术后两组临床结果数据 The details of the postoperative outcome

Variables related to surgery	Video-assisted thoracoscopic surgery (VATS) group	Thoractomy group	*P*
Mean stay in-hospital (days)	13.14±1.95	19.72±7.55	0.632
Mean stay in the intensive care (hours)	12.85±2.79	16.09±4.45	0.848
Operating room time (minutes)	164.28±30.47	236.36±24.60	< 0.001
Blood transfusion (units)	2.28±0.75	4.18±2.08	0.036
Postoperative drainage (mL)	461.42±79.04	865.45±127.30	< 0.001
Postoperative drainagetime (days)	3.71±0.75	5.00±0.63	0.001

随诊时间为术后10个月至术后5年，其中开胸组为术后18个月至术后5年，腔镜组为术后10个月至术后36个月。4例患者死亡（开胸组3例，腔镜组1例），2例N2患者于2年内死亡。两组患者生存曲线见图 1，生存率无明显差异（*P*=0.187）。由于腔镜手术多数为近2年完成，随诊期限相对早期开胸组短，尚不能完全确定两组5年生存率总体水平差异。

**1:**
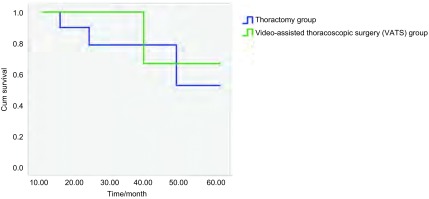
腔镜组与开胸组生存曲线 Survival curve between video-assisted thoracoscopic surgery (VATS) group and thoractomy group

## 3 讨论

目前对于肺癌的治疗强调以外科手术为主的综合治疗，肺切除加系统纵隔淋巴结清扫是肺癌首选的治疗手段。对于合并严重冠心病的患者，心肌缺血症状如果不被很好的纠正，肺癌外科治疗过程中麻醉的创伤、手术操作的损伤、围手术期的应激等因素都会导致严重的心肌梗塞的发生^[3, 4]^。内科介入治疗不适合三支冠脉血管或左冠脉主干病变的患者，支架植入后抗血小板治疗也增加了肺癌外科术中、术后出血的可能。肿瘤本身的高凝状态以及手术创伤直接影响植入支架的通畅率^[5]^。体外循环辅助下冠脉血管旁路移植手术对于严重冠心病是一种开展时间早、安全性和效果确切的治疗方法。由于体外循环的使用抑制体内细胞或体液介导的免疫系统，对抑制肿瘤生长起重要作用的自然杀伤细胞（natural killer cell, NK）的活性产生很大影响同时激活的炎性因子反应系统（inflammatory response syndrome, SIRS）也影响机体内环境的稳定^[6]^。因此对于严重冠心病合并肺癌的患者这种治疗方法的使用有待商榷。综合临床肺癌及冠心病的治疗原则，笔者认为肺癌根治手术联合非体外循环下不停跳冠脉旁路移植术对这类复合疾病患者是合理的治疗方案。

肺癌根治及不停跳冠脉搭桥手术是临床上两类成熟的术式，国外文献有同期手术报告，在充分和患者沟通征得同意后实施同期心肺手术符合临床治疗原则，无医学伦理问题。冠脉搭桥与肺癌根治手术同期实施，避免了分期手术带给患者两次麻醉、两次手术创伤以及住院时间、医疗费用增加等不良影响。但同期手术中肝素的使用增加了肺部手术出血的风险，同时经正中切口显露肺门及纵隔结构困难增加了肺癌根治手术的困难^[7, 8]^。为提高同期手术的安全性，早期病例在正中切口完成冠脉搭桥术后重新摆放患者体位，经后外侧胸部切口行标准肺叶切除及系统淋巴清扫。手术操作成熟尤其对外科技术要求较高的术式优势明显（开胸组中完成2例肺袖式切除，1例肺动脉成型）。但两个切口的损伤及疼痛以及延长的麻醉及手术时间使得患者术后心肺功能恢复较慢，围手术期并发症的发生率较高，本组病例早期的8例同期两切口操作的患者中术后发生肺不张2例，心律失常3例。近些年胸腔镜技术在胸外科手术中的应用越来越广泛。随着同期手术临床治疗经验的总结以及胸腔镜外科操作技术的积累，后期这类患者我们尝试在胸腔镜辅助下通过单一正中切口完成肺癌根治。正中切口完成肺叶切除最大的困难在于肺门及纵隔解剖视野的显露及操作，国外文献提示正中切口完成左侧肺叶切除尤为困难，胸腔镜的应用为我们提供了良好的操作视野，同时血管、气管切缝器等特殊器械的使用也使得肺叶切除相对容易。我们在冠脉旁路移植完成后，患侧抬高30度，于腋中线六肋及腋前线三肋水平放置胸腔镜及专用器械，目镜监测下，经正中切口由前到后单向推进方式逐一游离下肺韧带及肺叶动脉、静脉、支气管，以一次性腔镜切缝器处理所切除肺叶血管及支气管完成肺叶切除。肺癌根治手术中系统淋巴结清扫非常重要，标准淋巴结清扫对于术后准确病理分期及远期预后意义重大，配合单切口完成肺癌根治手术，我们在搭桥完毕心包关闭前，经升主动脉与上腔静脉间隙，右肺动脉下方打开心包后壁，显露支气管隆突清扫7号、4号淋巴结，肺叶切除后根据淋巴结不同解剖位置，胸腔镜辅助下行右侧2、3、8、9号或左侧5、6、8、9号淋巴结清扫，完成标准肺癌根治手术^[9]^。

肺癌根治和冠脉搭桥手术是两类创伤比较大的手术，同时实施中出现的心肺功能的应激对患者术后恢复影响很大，在麻醉的管理、外科操作的要求、围手术期心肺功能的支持等方面也有较高要求。胸腔镜手术以其外科操作创伤微小、术后恢复快、合并症减少等优点符合现代外科治疗的新理念。同时多中心病例统计^[10, 11]^显示，腔镜外科在早期肺癌的治疗中，无论在肺切除范围还是在系统淋巴结清扫等方面均与常规开胸肺癌根治手术无差异，远期治疗效果确切、可靠。由于本组病例数量有限，许多问题有待深入研究，综合现有的临床经验，对于肺部肿瘤，TNM分期Ⅰ期/Ⅱ期患者手术治疗效果较好，结合PET或纵隔镜检查考虑N2的患者，由于远期治疗效果不佳，应当排除于同期手术治疗适应症之外。术前判断肿瘤本身和周围血管、组织局部粘连密切需行支气管袖式切除或肺动脉成型等手术，胸腔镜下操作困难，侧开胸完成手术相对安全。同期手术创伤较大，对患者术前心肺功能要求较高，肺功能较差（VC < 60%, FEV_1_/FVC < 55%, FEV_1_ < 1.2 L, PO_2_ < 60 mmHg）或心脏结构及功能明显受损（EF < 35%、室壁运动明显异常合并室壁瘤形成、30天内新发心肌梗塞、血流动力学不稳定）等情况不适合肺癌根治联合搭桥手术同期治疗^[12]^。

对于肺癌合并严重冠心病的患者，同期肺癌根治及冠状动脉旁路移植手术安全、有效。胸腔镜辅助下完成肺癌根治手术创伤小，治疗效果确切。
